# Are vector-borne pathogen co-infections complicating the clinical presentation in dogs?

**DOI:** 10.1186/1756-3305-6-97

**Published:** 2013-04-15

**Authors:** Anna Sara De Tommasi, Domenico Otranto, Filipe Dantas-Torres, Gioia Capelli, Edward B Breitschwerdt, Donato de Caprariis

**Affiliations:** 1 Department of Veterinary Public Health, Faculty of Veterinary Medicine, University of Bari, Str. prov. per Casamassima km 3, Valenzano, Bari, 70010, Italy; 2 Departamento de Imunologia, Centro de Pesquisas Aggeu Magalhães, Fundação Oswaldo Cruz – Pernambuco, Av. Prof. Moraes Rego s/n, Recife, PE, 50670-420, Brazil; 3 Istituto Zooprofilattico Sperimentale delle Venezie, Viale dell’Università, Legnaro, Padova, 10, 35020, Italy; 4 Intracellular Pathogens Research Laboratory, Center for Comparative Medicine and Translational Research, College of Veterinary Medicine, North Carolina State University, Raleigh, North Carolina, USA

**Keywords:** Co-infection, *Dirofilaria immitis*, *Ehrlichia canis*, *Leishmania infantum*, Bone marrow

## Abstract

**Background:**

Infection by two or more canine vector-borne disease (CVBD)-causing pathogens is common in subtropical and tropical regions where vectors are plentiful. Co-infections may potentiate disease pathogenesis, thereby altering clinical manifestations typically associated with singular infections. These factors complicate diagnosis, treatment and can adversely influence prognosis if the practitioner fails to suspect, document, and treat each concurrent infection. The spectrum of pathogens co-infecting dogs may change over time in a given practice location due to the rapid expansion of arthropods and their associated vectored agents, and international transit among pets and wild animals. This applies, for example, to *Dirofilaria immitis* and *Leishmania infantum*, the distributions of which have expanded from northern to southern Italy, and vice versa, respectively. Indeed, mixed infections by *D. immitis* and *L. infantum* have only been reported once in Italy, probably due to the fact that competent vectors for these infections do not usually occur in the same geographical areas. Thus, information that would help practitioners to identify clinical presentations in dogs co-infected by *D. immitis* and *L. infantum* and other CVBD-causing pathogens is scant.

**Findings:**

This manuscript describes the clinical history and physical examination of findings for 7 CVBD co-infected dogs that were examined because of a spectrum of clinical signs. Five dogs were co-infected with *L. infantum* and *Ehrlichia canis*, one dog with *L. infantum*, *E. canis* and *D. immitis* and the remaining dog with *L. infantum* and *D. immitis*.

**Conclusions:**

The clinical signs and haematological abnormalities associated with the diagnostic evaluation and treatment of these dogs is discussed. Also, the usefulness of bone marrow specimens for the molecular diagnosis of CVBDs and for the enhanced monitoring of treatment response is emphasized.

## Findings

Canine vector-borne diseases (CVBDs) are caused by a spectrum of pathogens that are transmitted by arthropods, including ticks, fleas, mosquitoes and phlebotomine sand flies [1]. Dogs are reservoir hosts for several arthropod-borne pathogens, some of which are of major zoonotic concern [2]. In Italy, dogs may be infected by several CVBD-causing pathogens, such as *Leishmania infantum*, *Ehrlichia canis*, *Babesia* spp., *Bartonella* spp., *Cercopithifilaria* spp., *Hepatozoon canis*, *Anaplasma platys*, *Dirofilaria immitis* and *Dirofilaria repens*[3]. Canine leishmaniosis (CanL) and canine monocytic ehrlichiosis (CME) are both severe systemic diseases and each can be characterized by a wide range of overlapping clinical signs (e.g., lymph node enlargement, weight loss, and splenomegaly). Dogs with CanL may also present skin alterations (alopecia, furfuraceous dermatitis, ulcers, and nodular lesions) [4], whereas dogs infected with *E. canis* may display haemorrhagic disorders [5]. Heartworm disease, caused by *D. immitis,* may be an asymptomatic infection or may be associated with pulmonary and cardiovascular illnesses (e.g. cough, dyspnoea, exercise intolerance, ascites, renal failure, and lethargy), with severity of disease mostly related to the nematode burden [6]. Molecular assays, serological testing and cytology performed on different biological tissues (bone marrow and lymph node for CanL and blood for CME, blood for dirofilariosis) are the most frequently used tests for diagnosing the respective infections [7,8]. Recommended treatment for CanL is a combination of meglumine antimoniate or miltefosine with allopurinol [9], doxycycline for CME [10], and melarsomine dihydrochloride as adulticide treatment for dirofilariosis, followed by macrocyclic lactones as larvicide [11].

In subtropical and tropical regions where vectors are plentiful, co-infections may potentiate disease pathogenesis, thereby altering clinical disease manifestations typically associated with singular infections. These factors complicate diagnosis, treatment and can adversely influence prognosis if the practitioner fails to suspect, document and treat each concurrent infection [12-16]. To the authors’ knowledge, co-infection with *D. immitis* and *L. infantum* has only been reported in a single dog from Italy [17], probably due to the fact that the geographical distribution of the vectors for these infections does not usually overlap. Under the above circumstances, information regarding the clinical presentation of dogs co-infected by *D. immitis* and *L. infantum* is scant, thus representing a challenge for practitioners in their attempt to diagnose such conditions.

This manuscript describes the clinical history of seven dogs that were examined due to highly variable disease presentations. Co-infection with two or more CVBDs was diagnosed in all seven dogs.

From January to December 2011, 7 dogs were referred to the teaching hospital of The Department of Veterinary Medicine (University of Bari, Italy) for diagnostic evaluation (Table 1). Dogs were of different breeds, both sexes, and various ages and came from an area of southern Italy that is endemic for *L. infantum* and *E. canis*[3]. At admission, one dog (no. 5) had a severe tick infestation, whereas the other dogs were not infested at the time of examination, but had no history of acaricide treatment. When referred, clinical signs included depression (2/7), weight loss (2/7), hyperthermia (1/7), anorexia (3/7), exercise intolerance (1/7), pale mucous membranes (2/7), lymph node enlargement (5/7), tongue ulcers (1/7), furfuraceous dermatitis (1/7), ulcerative granulomatous dermatitis of prepuce (1/7), cough (1/7), lameness (1/7), epistaxis (1/7) and multiple mammary nodules (1/7). Complete blood counts (CBC) were obtained using an automated cell counter (Abbott Cell-Dyn 3700). Serum proteins were determined by agarose gel electrophoresis. The following haematological and serum biochemistry parameters were recorded: haemoglobin concentration (Hb), haematocrit (Hct), nucleated red blood cells (nRBC), white blood cells (WBC), platelet count (PLT), total serum protein (TP), albumin and γ-globulin. Haematological reference ranges were previously determined [18]. Blood, buffy coat, lymph node, and bone marrow smears were prepared and stained using the MGG Quick Stain (Bio Optica Spa, Italy) and stained-smears were examined by light microscopy for the presence of intracellular inclusions (or free forms) of common tick-borne pathogens and *L. infantum*[19]. All dogs had a polyclonal gammopathy. Anaemia and thrombocytopenia were found in five and four dogs, respectively. Cytological examination of lymph node and bone marrow smears resulted in the identification of *L. infantum* amastigotes in 6/7 dogs (nos. 1, 3, 4, 5, 6, and 7). In dogs 1 and 2, nematode microfilariae, identified as *D. immitis,* were initially confirmed by direct examination of blood smears and modified Knott’s test [20]. *Dirofilaria immitis* infection in these two dogs was further confirmed by the detection of circulating antigens using a commercial assay (SNAP heartworm antigen test; IDEXX Laboratories, Milano, Italy) and by PCR specific amplification of *Dirofilaria* spp. DNA, that was performed as described elsewhere [21]. PCR on blood and bone marrow samples was also performed for detection of *L. infantum*, *Ehrlichia*, *Anaplasma* and *Babesia* species, as previously described [19]. Five dogs were co-infected with *L. infantum* and *E. canis*, one dog with *L. infantum*, *E. canis* and *D. immitis* and the remaining dog with *E. canis* and *D. immitis*. The finding of microfilariae of *D. immits* in the bone marrow cytology was most likely due to the presence of blood in the bone marrow sample even if microfilariae have rarely been found in the bone marrow of dogs [22].

**Table 1:** Breed, sex, age (in years), description of clinical signs, laboratory abnormalities and diagnostic test results for 7 dogs co-infected by two or more canine vector-borne diseases causing pathogens

**Dog no./breed**	**Sex**	**Age**	**Clinical signs**	**Laboratory abnormalities**	**Cytology**				**PCR**	
					**Blood**	**Buffy coat**	**Lymph node**	**Bone marrow**	**Blood**	**Bone marrow**
1/Fila Brasileiro	F	5.5	Weight loss, anorexia, exercise intolerance, pale mucous membranes, lymph node enlargement, furfuraceous dermatitis	Anaemia, polyclonal gammopathy, low PLT	*Diro*	–	*Leish*	*Leish*, *Diro*	*Ehrl, Diro*	*Ehrl, Leish*
2/Fila Brasileiro	M	6	Asymptomatic	Polyclonal gammopathy	*Diro*	–	–	*Diro*	*Ehrl, Diro*	*Ehrl*
3/Mongrel	F	5	Tongue ulcers, lymph node enlargement	Polyclonal gammopathy	–	–	*Leish*	*Leish*	*Ehrl*	*Ehrl, Leish*
4/English setter	M	9	Ulcerative granulomatous dermatitis of prepuce	Anaemia, polyclonal gammopathy, low PLT	nd	nd	*Leish*	*Leish*	*Ehrl*	*Ehrl, Leish*
5/Siberian Husky	M	8	Coughing, anorexia, depression, hyperthermia, pale mucous membranes, lymph node enlargement	Anaemia, polyclonal gammopathy, low PLT	–	–	*Leish*	*Leish*	*Ehrl*	*Ehrl, Leish*
6/Doberman	F	4	Depression, weight loss, lymph node enlargement, multiple mammary nodules	Anaemia, hyperproteinemia, polyclonal gammopathy, low PLT	–	–	*Leish*	*Leish*	*Ehrl*	*Ehrl, Leish*
7/Schnauzer	F	5.5	Lameness, anorexia, lymph node enlargement, epistaxis	Anaemia, polyclonal gammopathy	–	–	*Leish*	*Leish*	*Ehrl*	*Leish*

Abbreviations: *F* female; *M* male; *PLT* platelets; *nd* not done; *Diro Dirofilaria immitis*; *Leish Leishmania infantum; Ehrl Ehrlichia canis*.

Dogs co-infected by *L. infantum* and *E. canis* were treated with a combination of meglumine antimoniate (50 mg/kg/bid/SC) and allopurinol (10 mg/kg/bid/OS) [23] and with doxycycline (10 mg/kg/die/OS). Dogs 1 and 2 were also treated with melarsomine 2.5 mg/kg/IM for two days for heartworm infections [6].

After therapy with doxycycline, whole blood PCR was negative for *E. canis,* whereas *E. canis* DNA was amplified from the bone marrow in five dogs (nos. 1, 2, 3, 4, and 6), which had normal platelet counts but failed to reduce gammopathy. For these dogs, an additional four weeks of treatment with doxycycline (10 mg/kg/die OS) was given. After two weeks PCR assays were negative on both blood and bone marrow samples from all five dogs and remained negative when retested at 4 weeks post-treatment.

## Discussion

Clinical evaluation of the seven co-infected dogs described in this case series illustrates the diagnostic, therapeutic and management challenges imposed by the spectrum of CVBDs prevalent in southern Italy. These concurrent infections can induce clinical illness, as illustrated by dog 1, that was infected by *E. canis*, *L. infantum* and *D. immitis,* as compared to asymptomatic infection in dog 2, that was infected with *E. canis* and *D. immitis*. Co-infections with *E. canis* and *L. infantum* are frequently detected in the Mediterranean region [24] whereas to date, co-infection with *D. immitis* and *L. infantum* has only been reported in a dog from Italy [17]. The region from which both dogs originated is endemic for *L. infantum* and *E. canis*[3]. Documentation of two additional cases of heartworm disease indicates that *D. immitis* occurs in autochthonous foci in southern Italy (i.e., both dogs had lived in this area since they were puppies, and had no travel history), thus confirming the spread of this helminthic infestation in this region [25,26]. Undoubtedly, infection with two or more pathogens complicates the clinical presentation, which provides the basis for the veterinarian’s diagnostic and therapeutic recommendations. In addition, co-infection with multiple CVBDs impacts the severity of haematological abnormalities in dogs, and poses challenges in terms of therapeutic strategies to be applied to each individual patient [18]. For example, *L. infantum* may impair the cellular and humoral immune response of the host, which may favour the establishment or the reactivation of a pre-existing *E. canis* infection [27]. Moreover, *E. canis* causes a reduction in major histocompatibility complex of class II receptors, which could ultimately enhance the clinical progression of CanL [28].

Out of five dogs infected with *L. infantum,* only dog 1 had clinical signs suggestive of CanL (e.g., lymphadenopathy, dermatitis and onychogryphosis), whereas the laboratory abnormalities (normocytic, normochromic non-regenerative anaemia, thrombocytopenia, and gammopathy) in the remaining *L. infantum-*infected dogs overlapped with laboratory abnormalities associated with CME [19]. This suggests that when infection with multiple pathogens is involved, the clinical presentation might be unpredictable and specific clinical or haematological abnormalities cannot be definitively ascribed to a single pathogen. For example, the polyclonal gammopathy detected in all seven dogs in this case series might be the consequence of a chronic antigenic stimulation [29] caused either by *L. infantum*, *E. canis* or *D. immitis* or any combination of these three pathogens.

Diagnosis of single or multiple CVBDs should rely on epidemiological information, including travel history, on the clinical status of the dog and on a panel of laboratory diagnostic tests [19]. In the cases reported herein, a definitive diagnosis of co-infection required a combination of clinical suspicion in conjunction with documentation of abnormal laboratory findings, and diagnostic confirmation by microscopy, serology and PCR, preferably using both blood and bone marrow specimens when attempting to confirm the diagnosis of *E. canis* and *L. infantum* infections. Accordingly, the tissues most frequently used for the diagnosis of *E. canis* infections are blood and bone marrow [7,30]. Monitoring the response following doxycycline administration in dogs suffering from ehrlichiosis is pivotal in distinguishing persistently infected sick dogs unsuccessfully treated, from dogs that achieve clinical and haematological recovery, but remain infected as compared to those dogs that recover and clear the pathogen [10]. Although preliminary and limited in quantity, data herein reported indicates that under natural exposure conditions, PCR on bone marrow aspirates may be a reliable method for the evaluation of the treatment response in dogs with CME. In our experience, when infections with *E. canis* and *L. infantum* occur in dogs with severe clinical or haematological abnormalities, these infections should be treated simultaneously to improve the dogs clinical status, before treating for *D. immitis*.

## Conclusions

The atypical clinical signs and mild to severe haematological abnormalities described in these seven co-infected dogs highlight the importance of a holistic diagnostic approach that includes microscopy, serology and PCR testing, when dealing with CVBDs in endemic areas. This report also supports the potential utility of using bone marrow specimens as a biological tissue for the molecular diagnosis of CVBDs and for monitoring of the treatment response in dogs with CME. Our results further stress the need for continued development of multi-pathogen detection methods for CVDBs in endemic and non-endemic regions.

## Competing interest

The authors declare that they have no competing interests.

## Authors’ contributions

ASDT and DdC conceived the study and documented clinical cases. ASDT, DdC, DO, FDT, EBB and GC drafted and revised of manuscript. All authors read and approved the final version of manuscript.
